# Universal sensor array for highly selective system identification using two-dimensional nanoparticles†
†Electronic supplementary information (ESI) available: The results of additional experiments, characterization of 2D nanomaterials and their nanoassemblies, and tables for Partial Least Squares (PLS) discriminant analysis, (Fig. S1–S11). See DOI: 10.1039/c7sc01522d



**DOI:** 10.1039/c7sc01522d

**Published:** 2017-06-16

**Authors:** Mustafa Salih Hizir, Neil M. Robertson, Mustafa Balcioglu, Esma Alp, Muhit Rana, Mehmet V. Yigit

**Affiliations:** a Department of Chemistry , University at Albany, State University of New York , 1400 Washington Avenue , Albany , New York 12222 , USA . Email: myigit@albany.edu ; Tel: +1-518-442-3002; b The RNA Institute , University at Albany, State University of New York , 1400 Washington Avenue , Albany , New York 12222 , USA

## Abstract

A typical lock-and-key sensing strategy, relying only on the most dominant interactions between the probe and target, could be too limiting. In reality, the information received upon sensing is much richer. Non-specific events due to various intermolecular forces contribute to the overall received information with different degrees, and when analyzed, could provide a much more powerful detection opportunity. Here, we have assembled a highly selective universal sensor array using water-soluble two-dimensional nanoparticles (nGO, MoS_2_ and WS_2_) and fluorescent DNA molecules. The array is composed of 12 fluorescently silent non-specific nanoreceptors (2D-nps) and used for the identification of three radically different systems; five proteins, three types of live breast cancer cells and a structure-switching event of a macromolecule. The data matrices for each system were processed using Partial Least Squares (PLS) discriminant analysis. In all of the systems, the sensor array was able to identify each object or event as separate clusters with 95% confidence and without any overlap. Out of 15 unknown entities with unknown protein concentrations tested, 14 of them were predicted successfully with correct concentration. 8 breast cancer cell samples out of 9 unknown entities from three cell types were predicted correctly. During the assembly of each nanoprobe, the intrinsic non-covalent interactions between unmodified 2D nanoparticles and ssDNAs were exploited. The unmodified 2D materials offer remarkable simplicity in the layout and the use of ssDNAs as probes provides limitless possibilities because the natural interaction of a ssDNA and 2D surface can be fine-tuned with the nucleobase composition, oligonucleotide length and type of 2D nanomaterial. Therefore, the approach described here can be advanced and fine-tuned indefinitely for meeting a particular sensing criterion. Though we have only studied three distinct elements, this approach is universal enough to be applied to a wide-range of systems.

## Abstract 2

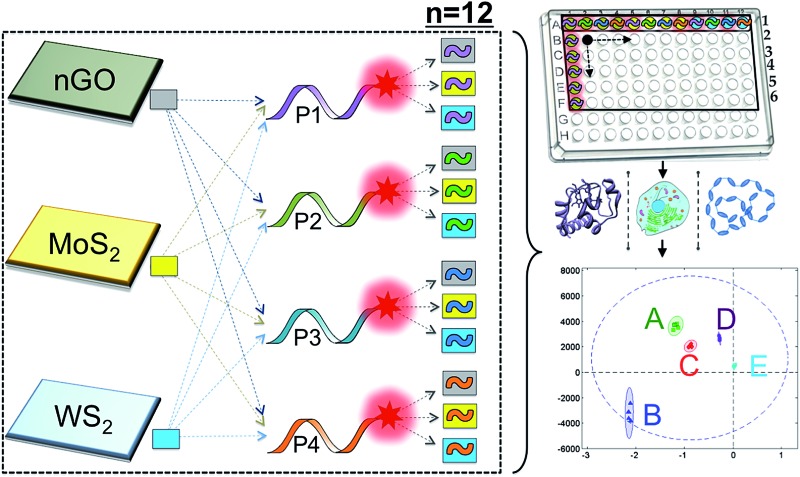
The universal sensor array is composed of 12 fluorescently silent non-specific artificial nanoreceptors (2D-nps) for the identification and classification of five proteins, three types of breast cancer cells and a structure-switching event of a macromolecule.

## 

A typical molecular recognition study is performed using a sensor which exploits the highly specific interactions between the probe and target molecules. Though this lock-and-key strategy enables sophisticated sensory designs, taking only the most dominant and highly specific interactions into account could be limiting. In reality, the information received upon sensing is much more detailed. Non-specific events due to various intermolecular forces contribute to the overall received information with different degrees, and in most of the cases, are too significant to ignore. When these non-specific interactions are discarded, the analyzed data could miss critical information which could offer a much more powerful multiplexed detection opportunity. Here, we have assembled a highly selective universal sensor array, which is composed of two-dimensional nanoparticles and DNA assemblies. The same sensor array was used for the identification of three radically different systems thanks to the non-specific interactions between the components of the sensor array and the target systems.

Nanotechnology has attracted significant attention in the last two decades.^1–7^ Recently, two-dimensional graphene-like materials have been studied extensively due to the distinct physical properties at their large 2D surfaces.^8–11^ Particularly nano-graphene oxide (nGO), a two-dimensional water-soluble carbon material, has been employed in a number of advanced applications.^12–16^ nGO is capable of highly efficient and exceptional single stranded (ss)DNA adsorption along with an ultrafast and super-efficient fluorescence quenching ability, both of which have been used for DNA-based sensing.^17–22^ Discovery of the exceptional properties of nGO motivated materials scientists to explore other 2D nanoparticles. Recently, 2D transition metal dichalcogenide (TMD) (*e.g.*, MoS_2_, WS_2_, *etc.*) nanosheets have emerged due to their graphene-like features.^23–35^ For instance, MoS_2_ and WS_2_ have attracted notable attention due to their affinity for ssDNA adsorption and fluorescence quenching capability.^30,36^ Though there are similarities in adsorption/desorption properties, different intermolecular forces contribute to each nanosheet and DNA assembly at varying degrees and ratios.^37^ We have exploited these differences in the non-covalent interactions to create a highly powerful universal non-specific sensor array.

DNA is a fascinating biopolymer providing versatile functionality to the nanomaterials due to its highly programmable features and unique interactions at the bio-nano interfaces.^38,39^ Synthesis of large-scale DNA molecules with various lengths and nucleobase compositions is facile, fast and cost-efficient. Motivated with its outstanding properties and ease of synthesis, DNA nanotechnology has been conducted in association with a broad range of applications.^40,41^ Oligonucleotides have been largely employed as aptamers or DNAzymes (functional oligonucleotides) to design target-specific biosensors.^42–44^ For instance, we have used 2D nanoparticle and ssDNA nanoassemblies for the simultaneous detection of circulating miRNAs or different types of biological or chemical compounds.^14,45^ However, these DNA-integrated 2D sensing platforms focus only on the strongest interaction, such as the hybridization event between the complementary probe and target oligonucleotides or structure switching upon specific molecular recognition. Such an approach however rules out all other interactions, such as the ones between the target and 2D surface, contributing to the overall received signal.

To overcome these challenges, here we have assembled a non-specific receptor array composed of DNA-adsorbed nGO, MoS_2_ and WS_2_ nanoparticles. The fluorescence sensor array is composed of 12 fluorescently silent non-specific artificial nanoreceptors (2D-nps) for the identification and classification of five proteins, three types of breast cancer cells and a structure-switching event of a macromolecule. The non-specific sensing approach is advantageous because it enables one to include every single; minor or major; transaction into the whole data set, providing comprehensive information about the target molecule.^46–55^ It also eliminates the concerns about background or false-positive signals, which could emerge from target-specific methods. Furthermore, the non-specific identification technique promotes a universal sensing platform for a wide variety of molecules and resolves the limitations arriving from the requirement of specific probe design for every single target molecule. In order to identify a series of analytes, array-based approaches offer important advantages compared to individual sensor designs. First, they enable multiple readings for a single molecule, which results in the collection of a great deal of data and provides more detailed information and characteristic response patterns to be analyzed by statistical methods. This strategy enhances the precision and reproducibility for the sensing technique without any bias. Second, the sensor array achieves a better discrimination between different targets, and its resolving power can always be improved by increasing the number of sensor elements.

## Results and discussion

**Scheme 1:**
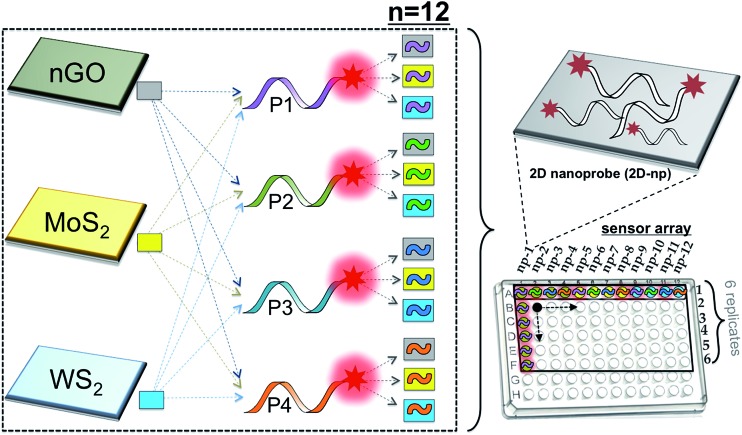
Sensor array constructed using 12 nanoprobe (2D-np) combinations. The 2D-nps were assembled *via* non-covalent interactions between three 2D nanoparticles (nGO, MoS_2_, WS_2_) and four FAM-labeled ssDNA molecules (P1, P2, P3 and P4). 2D-nps: np-1: [nGO-P1], np-2: [nGO-P2], np-3: [nGO-P3], np-4: [nGO-P4], np-5: [MoS_2_-P1], np-6: [MoS_2_-P2], np-7: [MoS_2_-P3], np-8: [MoS_2_-P4], np-9: [WS_2_-P1], np-10: [WS_2_-P2], np-11: [WS_2_-P3], np-12: [WS_2_-P4].

We have assembled a universal sensor array composed of 12 different nanoassemblies (2D nanoprobes: 2D-nps), which are prepared by the adsorption of four non-specific fluorescently labeled short oligonucleotides (FAM-scrambled, FAM-A23, FAM-C23, FAM-T23) on three 2D nanomaterials (nGO, MoS_2_ and WS_2_), Scheme 1. Each 2D nanomaterial was characterized using UV-visible spectroscopy for their characteristic absorbance bands and dynamic light scattering (DLS) for their hydrodynamic radius (∼100 nm) prior to the detection studies (Fig. S1†). The 2D nanomaterials were used as templates for constructing the artificial nanoreceptors (2D-nps). Each 2D-np in the sensor array was prepared by the adsorption of a FAM-labeled non-specific ssDNA probe on a 2D nanoparticle surface through a 30 min incubation in proper buffer conditions. The fluorescence of the probe DNA was quenched upon the adsorption on the 2D surface. Since each nanomaterial uses different combinations and types of intermolecular forces (van der Waals forces, H-bonding, π–π stacking, electrostatic interactions and *etc.*) with varying degrees and ratios for adsorption/desorption of the DNA probes, each 2D-np functions as a non-specific, yet unique, artificial receptor.^37,56^ The combination of these 12 2D-nps in a sensor array produces a large comprehensive data set which can be analyzed by statistical methods for non-specific, yet selective, target identification, Scheme 1.

**Fig. 1:**
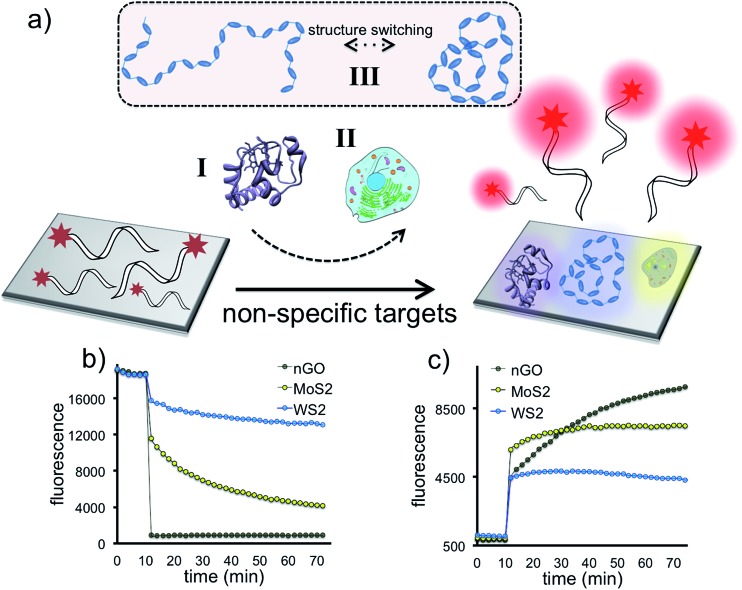
Adsorption, fluorescence-quenching and selective displacement of a fluorescent DNA probe on 2D nanoparticles (nGO, MoS_2_, WS_2_). (a) Illustration of the selective displacement event upon the introduction of three different systems. (b) Adsorption of a fluorescent DNA probe using a constant 2D nanoparticle concentration. (c) Desorption of a fluorescent DNA probe from three different 2D-nps by a constant concentration of an analyte.

The sensory system relies on the highly reproducible and unique desorption responses created by each 2D-np against a broad spectrum of targets varying from proteins to living cells to the dynamic conformational change of a thermo-responsive polymer, Fig. 1a. First, we investigated the quenching performance of each 2D nanoparticle against the same amount of a FAM-labeled DNA molecule, Fig. S2.† We incubated 20 nM of P2 molecule (FAM-A23) with 3.0 μg mL^–1^ of nGO, MoS_2_, or WS_2_ and monitored the quenching over an hour, Fig. 1b. Results indicate that for the same initial fluorescence, constant amounts of each 2D material displayed different degrees and rates of quenching. This could be due to the number, type, degree and ratio of the intermolecular forces taking place between the probe molecules and the nanoparticle surface.^37^ The greatest fluorescence-quenching event was observed with nGO followed by MoS_2_ and WS_2_, respectively. The MoS_2_ displaying a greater ssDNA adsorption capacity than WS_2_ could be because WS_2_ has a smaller surface area and is more negatively charged than MoS_2_, and thus has less favorable interactions with negatively charged ssDNA.^37^

Next, we studied the displacement of the surface-adsorbed DNA probes in the presence of a non-specific macromolecule. 1 μM of alkaline phosphatase was added into 100 μL of completely (∼95%) quenched [nGO-P4], [MoS_2_-P4], or [WS_2_-P4] 2D-np suspensions and fluorescence recovery was monitored until the reaction reached a plateau phase (2 h) for each 2D-np. For a constant protein concentration, different fluorescence recovery responses are received for the same FAM-labeled DNA probe following two-step kinetics, Fig. 1c. The differential desorption degree and rate could be due to the distinct interactions of (a) the protein with the nanosheet surface or (b) the DNA probe with the nanosheet surface or a combination of both. Nevertheless, the protein competes with the pre-adsorbed DNAs and displaces a certain amount resulting in a unique fluorescence recovery response with each 2D-np. This initial test, using only one model analyte and three 2D-nps, suggests that the elements of our sensor array enable one to collect differential information for the same target molecule and provide a more comprehensive and informative glance at the data for non-specific identification of a wide spectrum of target molecules.

**Fig. 2:**
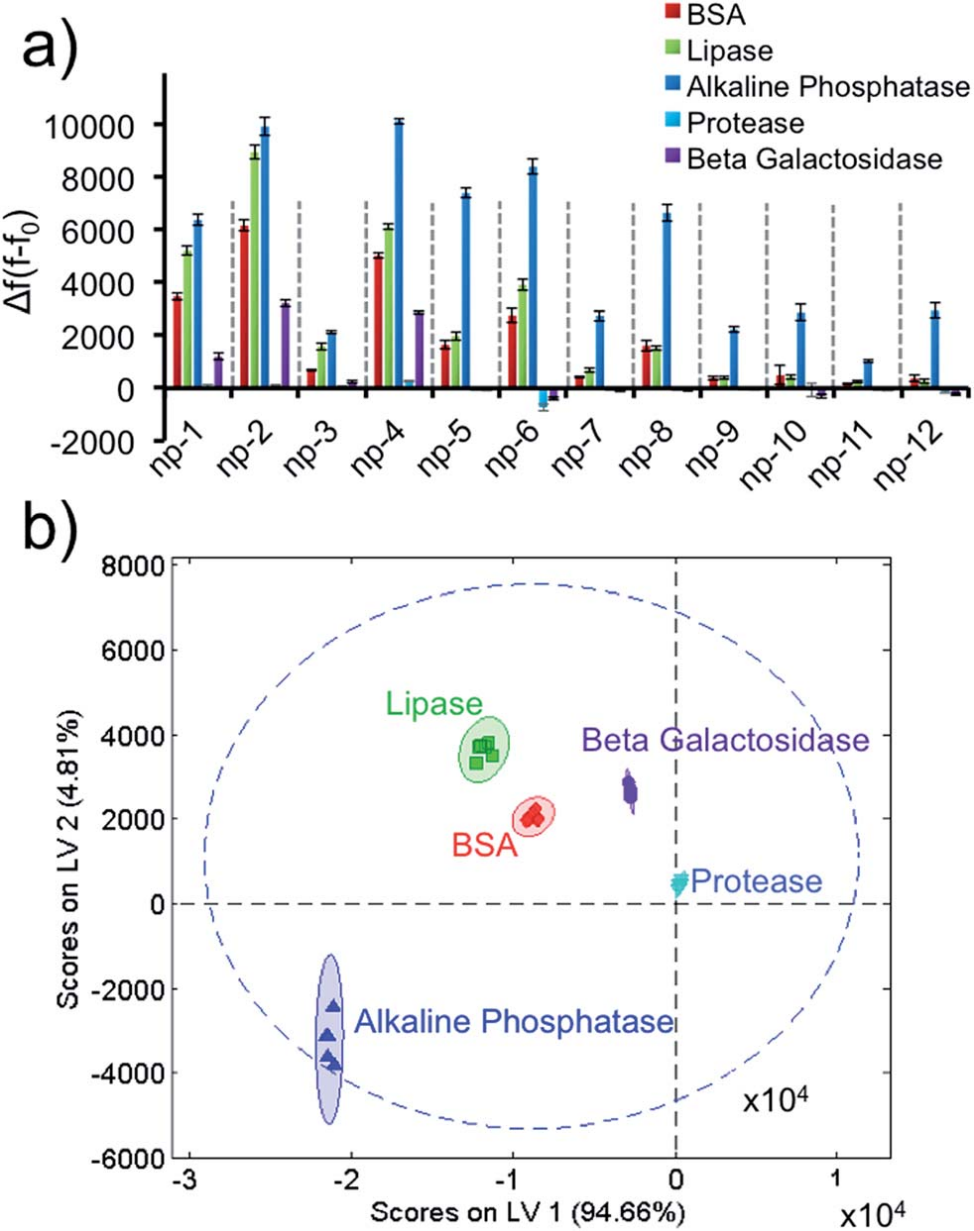
Protein identification using the non-specific sensor array. (a) Fluorescence response patterns of the sensor array (np1–np12) against 1 μM of proteins (BSA, lipase, alkaline phosphatase, protease, β-galactosidase). (b) Canonical score plot for the first two latent variables of processed fluorescence response patterns obtained against 1 μM proteins. The canonical scores were calculated by PLS for the identification of five proteins. All five proteins were well-discriminated and identified accurately with 95% confidence.

Next, we identified five different proteins using unique responses received by the elements of the sensor array for each protein. First, 1 μM target proteins (BSA, lipase, alkaline phosphatase, protease, β-galactosidase) were tested against the sensor array in six replicates. For each 2D-np, the final fluorescence at the end of a 2 hour kinetics study was used to calculate the fluorescence increase, Δ*f*(*f* – *f*_0_), which occurred upon the displacement of the probe molecules by target proteins. These fingerprint data were put together for the whole sensory system to obtain a training matrix (5 proteins × 12 nanoprobes × 6 replicates) to construct the fluorescence response patterns against each protein (Fig. 2a and S3†). Results indicate that the 2D-nps generate unique and highly reproducible responses against the proteins. In order to visualize the data matrix, the raw data were processed *via* Partial Least Squares (PLS) discriminant analysis to identify each protein (Fig. 2b). PLS enables one to differentiate two or more groups of objects or incidents by determining the features that best describe the differences within or between the groups. Results reveal that our sensor array was able to identify five proteins as separate clusters with 95% confidence and without any overlap. In our studies, we chose 23 nt long ssDNAs (P1: scrambled, P2: poly-A, P3: poly-C and P4: poly-T) which provided differential and unique responses with each 2D-np and target group. In all cases, the 2D-nps with surface-adsorbed poly-C (P3) were more resistant to desorption than their counterparts.^57^

**Fig. 3:**
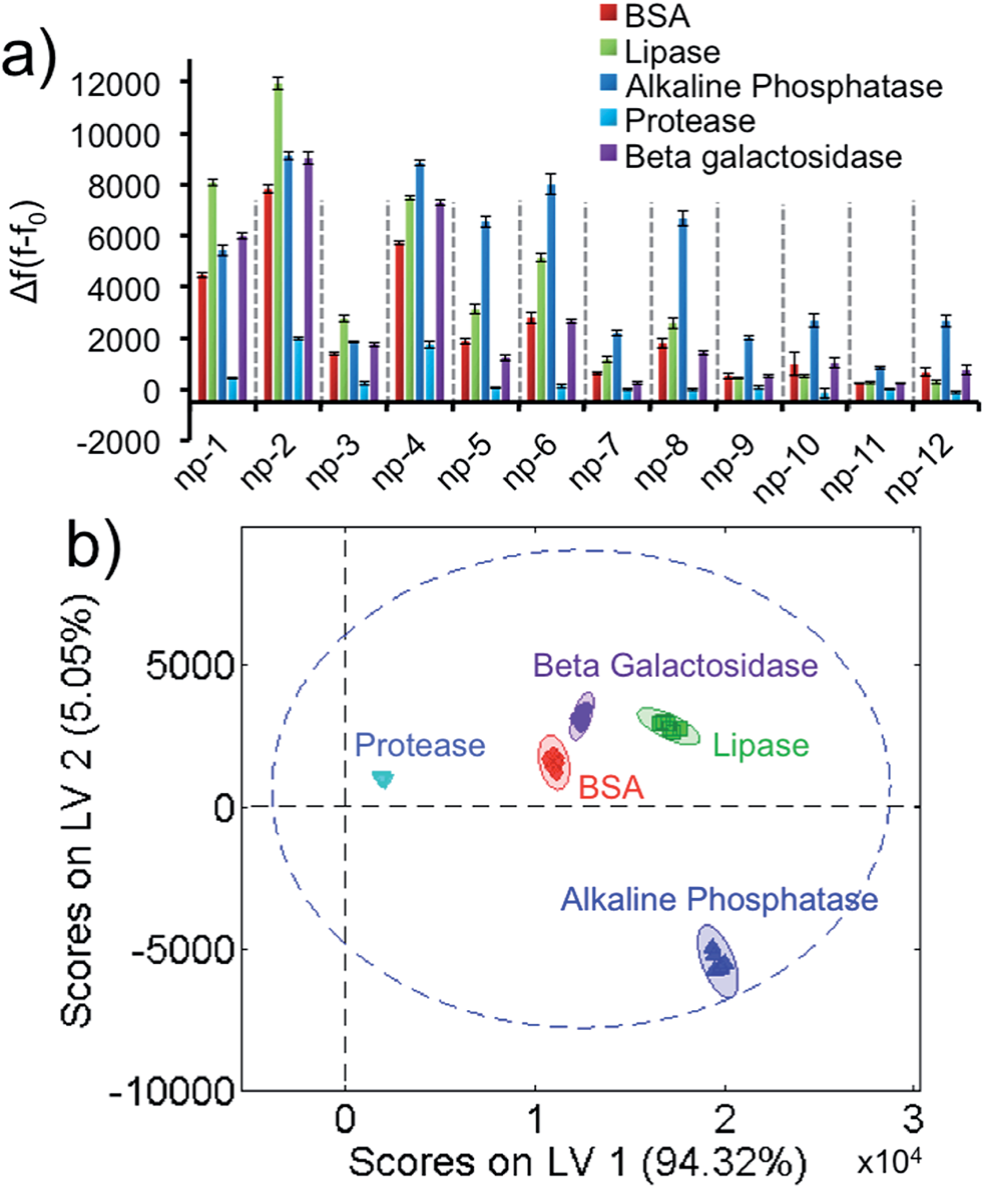
Protein identification using the non-specific sensor array. (a) Fluorescence response patterns of the nanoprobe sensor array (np1–np12) against proteins with fixed absorbance value (*A*_280_ = 0.1 a.u.). (b) Canonical score plot for the first two latent variables of processed fluorescence response patterns obtained against proteins with identical absorbance. The canonical scores were calculated by PLS for the identification of five absorbance-normalized proteins. All five proteins were well-discriminated and identified accurately with 95% confidence.

While differentiating 5 proteins with a fixed concentration is interesting from a fundamental standpoint, this data set is unable to determine an unknown protein unless its concentration is 1 μM. Ideally a sensor array should be able to identify an unknown protein with an unknown concentration. In order to address this limitation, we performed PLS discriminant analysis by normalizing the absorbance of each protein to an identical absorbance value. This UV-visible spectroscopy-coupled approach offers a calibration plot for the identification of a protein and its concentration when neither are known. In order to normalize any unknown protein sample, we decided on a constant absorbance value and produced the fluorescence response patterns for the protein amounts corresponding to 0.1 a.u. at 280 nm (*A*_280_ = 0.1 a.u.). The 0.1 a.u.-equivalent protein concentrations were used as standards for our sensor array. The data matrix was obtained from 2D-nps against the proteins (5 proteins × 12 nanoprobes × 6 replicates) and processed to produce the fluorescence response patterns (Fig. 3a and S4†). Results demonstrate that 0.1 a.u.-equivalent protein concentrations result in unique fingerprints in a highly reproducible fashion as well. The training matrix was subjected to PLS to identify each standard protein sample in separate clusters (Fig. 3b), which enabled us to differentiate each protein with 95% confidence without any overlap.

Since our calibration plots were ready for each protein, we challenged our non-specific sensor array against 15 different entities composed of unknown proteins with unknown amounts. First, each sample was analyzed by UV-visible spectroscopy to determine the initial absorbance values. Then, the absorbance numbers were normalized to 0.1 by applying the necessary dilution to the initial concentrations. The dilution coefficient for each sample was recorded for upcoming back-calculations. The normalized 0.1 a.u.-equivalent concentrations were tested against the sensor array in six replicates. The resulting data matrix for 15 samples (15 unknowns × 12 nanoprobes × 6 replicates) was processed by the PLS prediction function and the discriminant analysis resulted in 14 successful predictions (93% agreement). Once the protein types were identified by PLS, the original concentrations were back-calculated using the dilution coefficient recorded initially for each unknown, Table 1. This result validates that our sensor array enables one to identify an unknown protein accurately and calculate its original concentration.

**Table 1:** Identification of unknown proteins and concentrations using PLS prediction function

Sample #	Identification			Verification	
	Protein	Abs_280_	Conc.	Protein/Conc.	Deviation
1	Lipase	0.018	6.8 μM	Lipase/6.8 μM	0%
2	Lipase	0.056	21.3 μM	Lipase/20.4 μM	+4.2%
3	Protease	0.062	27.3 μM	Protease/27.8 μM	–1.8%
4	ALP	0.042	2.8 μM	ALP/3.1 μM	–9.7%
5	Lipase	0.027	10.3 μM	Lipase/10.2 μM	+1.0%
6	Protease	0.037	16.3 μM	Protease/18.5 μM	–11.9%
7	BSA	0.023	6.7 μM	BSA/7.0 μM	–4.3%
8	BSA	0.035	10.2 μM	BSA/10.5 μM	–2.6%
9	BSA	0.075	21.8 μM	BSA/21.0 μM	+3.7%
10	Protease	0.026	11.4 μM	Protease/13.9 μM	–18.0%
11	Protease	0.124	54.6 μM	Protease/55.6 μM	–1.8%
12	β-GAL	0.029	9.3 μM	β-GAL/9.2 μM	+1.1%
13	ALP	0.026	1.7 μM	ALP/2.1 μM	–19.1%
14	ALP	0.080	6.4 μM	ALP/6.2 μM	+3.1%
15	BSA	0.016	3.5 μM	β-GAL/4.6 μM	Fail

**Fig. 4:**
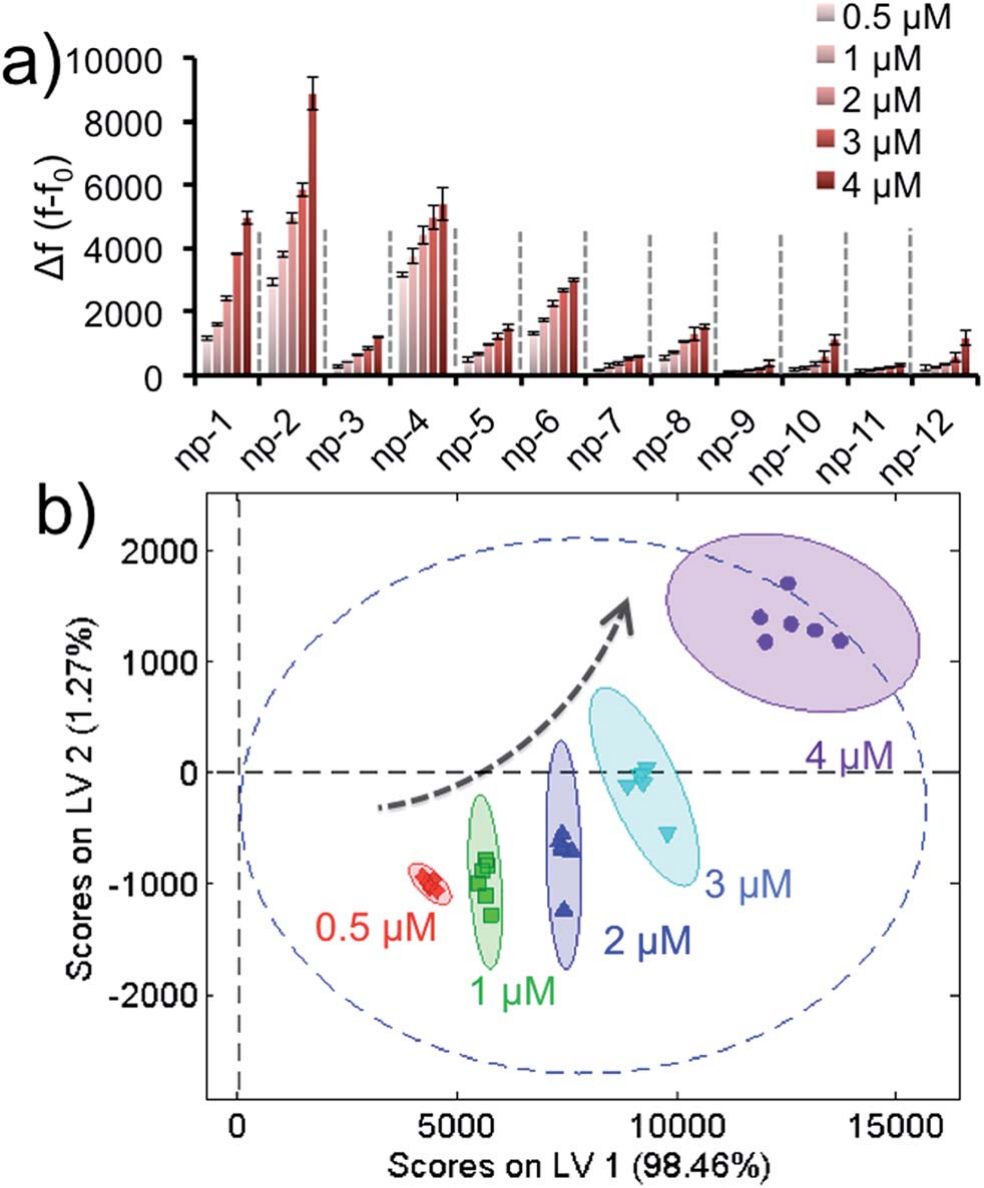
Concentration-dependent response of the sensor array. (a) Fluorescence response patterns of the nanoprobe sensor array (np1–np12) against various protein concentrations. (b) Canonical score plot for the first two latent variables of processed fluorescence response patterns obtained against five different concentrations. The canonical scores were calculated by PLS for the determination of five protein quantities. All five concentrations were well-discriminated and identified accurately with 95% confidence.

As a separate note, it was important to show that the fluorescence recovery responses were concentration-dependent, which would validate our aforementioned unknown test. In order to demonstrate that different target concentrations could generate well-separated clusters in PLS data, various concentrations (0.5 μM, 1.0 μM, 2.0 μM, 3.0 μM or 4.0 μM) of a non-specific protein were tested with the sensor array. The training matrix was formed to produce the fluorescence response patterns, Fig. 4a and S5.† The data was analyzed by PLS and the score plot was obtained with 95% confidence and without any overlap, Fig. 3b. Results reveal that different concentrations of a target generate different clusters in the score plot; thus, our approach using concentration normalization in Fig. 3b was indeed required for unknown protein identification. In addition, the results also suggest that the sensory approach was able to classify a protein according to its concentration within a nanomolar range.

In order to demonstrate the universality and discrimination power of our approach, we elaborated our sensory array for a much more complex target group. We challenged our design to identify three distinct human breast cancer cell types. Two triple-negative breast cancer (TNBC) cell lines; highly metastatic MDA-MB-231 and non-metastatic BT-20 cells; were tested along with non-metastatic MCF-7 cells which, unlike the previous two, express estrogen and progesterone receptors (ER^+^ and PR^+^). While MCF-7 cells are categorized under the luminal A subtype, TNBC cells are classified under the basal subtype and are considered much more aggressive and difficult to treat.^58,59^ Therefore, there are commonalties and differences among all three cells in terms of cell surface signatures, subtypes, disease progression and aggressiveness. The differentiation of subtypes is significant for clinical purposes because the identification of the specific disease type could enable critical prognostic and therapeutic implications for breast cancer patients.^60^ These multi-layered target profiles present a critical task to differentiate each cell type.

**Fig. 5:**
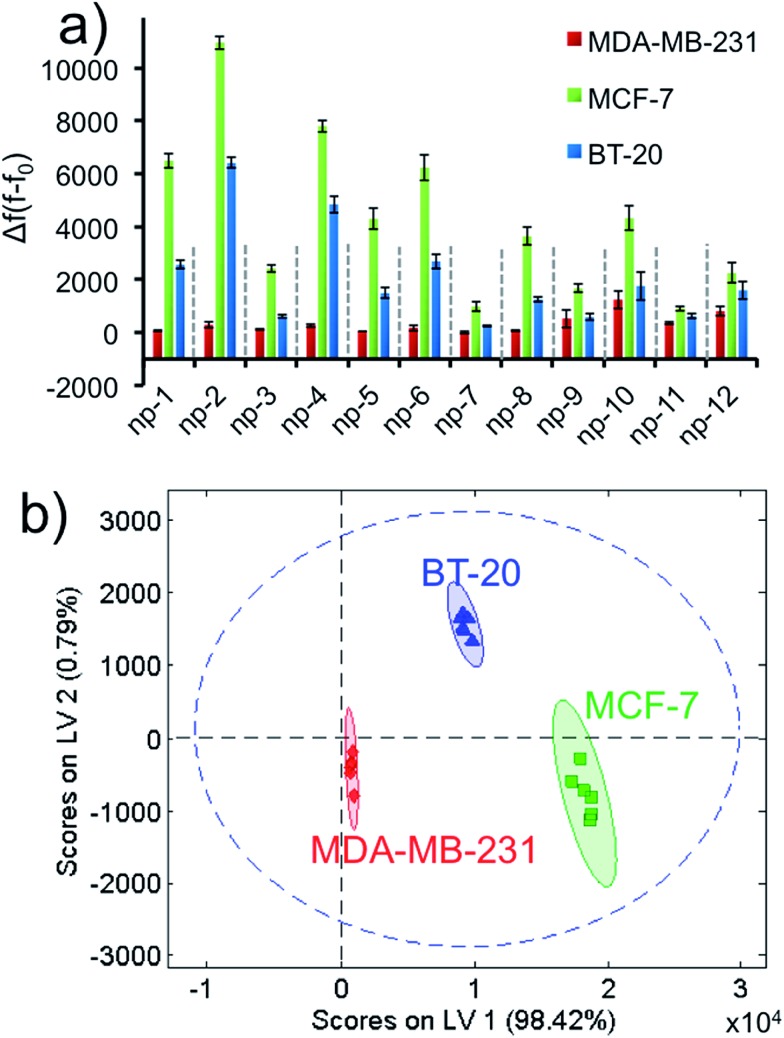
Breast cancer cell identification using the non-specific sensor array. (a) Fluorescence response patterns of the nanoprobe sensor array (np1–np12) against different cell lines (MDA-MB-231, MCF-7, BT-20) with constant cell number (∼1000). (b) Canonical score plot for the first two latent variables of processed fluorescence response patterns obtained against the cell types. The canonical scores were calculated by PLS for the identification of three cell lines. All three cell types were well-separated and identified accurately with 95% confidence.

We ran the sensor array to identify three different non-specific breast cancer cells with a constant cell number. After growing the cells under proper conditions, the cells were suspended in phosphate buffer. Each 2D-np in the sensor array was challenged against ∼1000 cells over 2 hours. Based on the fluorescence recovery from the DNA displacement by cancer cells, the training matrix was produced to construct the fluorescence response patterns, Fig. 5a and S6.† Results reveal that MCF-7 cells displace the greatest number of probe molecules from the surface, while MDA-MB-231 cells displace the least. These unique responses provided a fingerprint allowing us to easily classify each cell type with PLS analysis.

Even though there is variability in size and morphology within the same cell line, all of these parameters are averaged out and factored in our data set. Thus, we were unambiguously able to discriminate each cell type using the canonical score plot which separated the clusters with 95% confidence (Fig. 5b).

Next, we challenged our sensor design to identify 9 different unknowns prepared from any one of the three cell types. The studies were carried out for ∼1000 cells. The data matrix was obtained by recording fluorescence increase for each 2D-np. The data were analyzed *via* PLS prediction function. Using the calibration data that we had already obtained from the standard cells, 8 unknowns out of 9 were predicted accurately (Table 2). The results indicate that we can identify as little as ∼1000 complex biosystems making our design one of the most sensitive sensor arrays for cell classification.^47,54,61–63^

**Table 2:** Identification of unknown cell types using PLS prediction function

Sample #	Identification	Verification
	Cell type	Cell type
1	MDA-MB-231	MDA-MB-231
2	BT-20	BT-20
3	MDA-MB-231	MDA-MB-231
4	BT-20	MCF-7 (fail)
5	MCF-7	MCF-7
6	BT-20	BT-20
7	MDA-MB-231	MDA-MB-231
8	MCF-7	MCF-7
9	BT-20	BT-20

**Fig. 6:**
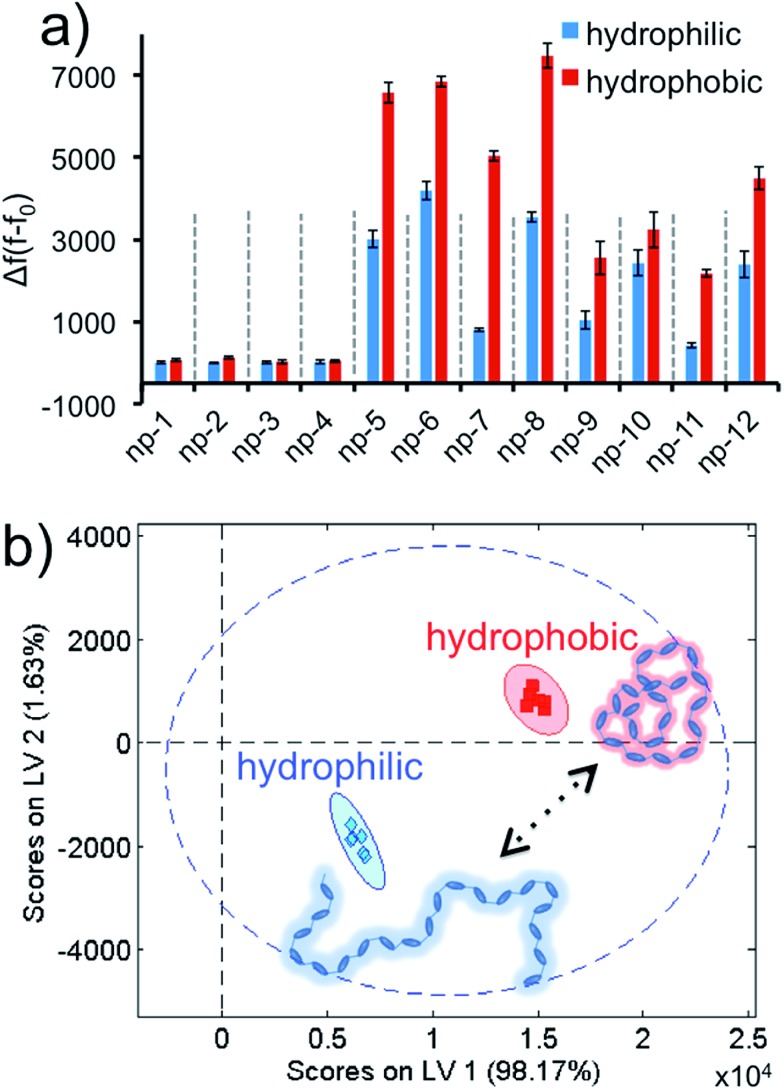
Conformation identification for a macromolecule using the non-specific sensor array. (a) Fluorescence response patterns of the nanoprobe sensor array (np1–np12) against thermo-responsive PNIPAM below and above the LCST (hydrophilic/hydrophobic, respectively). (b) Canonical score plot for the first two latent variables of processed fluorescence response patterns obtained against distinct conformations of the polymer. The canonical scores were calculated by PLS for the identification of two solubility states. Molecular configurations were well-separated and identified accurately with 95% confidence.

Later, we demonstrated that our approach was not only advanced enough to identify different biomolecules or living systems, but also was highly sophisticated for the detection of a dynamic phase transition of a macromolecule. We challenged our sensor array against poly(*N*-isopropylacrylamide) (PNIPAM), a thermo-responsive polymer with a lower critical solution temperature (LCST) of 39 °C, at two different conformations. PNIPAM has a sharp phase transition around its LCST, and displays a hydrophilic character below and hydrophobic state above its LCST.^64^ The experiments were carried out at two temperatures, below and above the LCST, in a sequence by using PNIPAM (0.037 mg mL^–1^ final) in six replicates. The acquired Δ*f*(*f* – *f*_0_) values were brought together to form the data matrix (2 conformations × 12 nanoprobes × 6 replicates) to obtain the fluorescence response patterns, Fig. 6a and S7.† The two phases of the PNIPAM resulted in discriminative responses. These fingerprint patterns were visualized and transformed into the score plot using the PLS method (Fig. 6b). Results demonstrate that different structural modes of PNIPAM can be identified by our non-specific universal sensor array with 95% confidence and without any overlap. Finally, in order to evaluate whether the 2D-nps were stable with the two temperatures that the aforementioned studies were performed at, we tested the sensor array at either temperature in the absence of any target, Fig. S8.† As predicted for this control experiment, the 2D-nps were stable at both temperatures without any false positive signal responses, which suggest that the received data set using PNIPAM was due to the phase transition of the polymer.

## Conclusion

Due to its remarkable differentiation strength, combined with a powerful statistical discriminant analysis method, the universal sensor array described herein offers a great means of non-specific identification for targets. Each non-specific nanoreceptor (2D-np) in the sensor array employs fluorescently labeled ssDNA molecules and water-soluble 2D nanomaterials. Unlike other related methods published in the literature, our approach described here is highly practical and has unlimited possibilities for the assembly of the sensory elements.^46–54^ During the nanoprobe assembly, we benefited from natural non-covalent interactions between nanoparticles and ssDNAs. Previous reports exploit the introduction of artificial interactions through synthetic procedures which could be time-consuming, costly and challenging under different circumstances.^46–48,65,66^ The unmodified 2D materials however offer remarkable simplicity in the layout in comparison with the surface modified nanoparticles.^46–48,65,66^ Moreover, the covalent modifications in the construction of a sensor array may limit the number of resulting sensing probes. On the other hand, because the interaction of the ssDNA and 2D surface can be fine-tuned with the nucleobase composition and length, each DNA acts as a unique probe providing a great diversity and limitless opportunity. Furthermore, labeling the DNA with low-cost fluorescent dyes from a large fluorophore library allows us to overcome the restrictions of using the limited number of fluorescent proteins or polymers. Besides, different types of 2D nanomaterials, including 40-some natural TMDs, could be used as a template for assembly of the nanoreceptors.^67^ Therefore, the approach described here can be advanced and fine-tuned indefinitely for meeting a particular criterion. Though using a single 2D nanoparticle could be somewhat useful for identification of a narrower target pool,^54^ inclusion of three different 2D nanoparticles, maybe even more, and different DNAs maximizes the chemical space that can be probed, Fig S9–S11.†

In conclusion, the proposed sensor array provides comprehensive and reliable information with a high reproducibility by catching even trivial events taking place between the platform and the target molecules. This sensory system was used to identify not only different targets with different physical, chemical or structural properties but also different states of the same target group. Our sensor array was able to identify various proteins with distinct molecular properties and living cancer cells with subtle differences. The sensory array was able to detect and discriminate as little as ∼1000 cells demonstrating greater sensitivity than previous reports.^47,54,61–63^ Last but not least, we could distinguish structural alterations of a smart thermo-responsive macromolecule that responds to a change in the environment. Though we have only studied three distinct elements, this approach is universal enough to be applied to a wide-range of systems.

## Materials and methods

All DNA sequences were purchased from Integrated DNA Technologies (IDT), USA with the following sequence information,

(P1) FAM-labeled scrambled DNA, 5′-/56-FAM/TCAACATCAGTCTGATAAGCTA-3′

(P2) FAM-labeled A23 DNA, 5′-/56-FAM/AAAAAAAAAAAAAAAAAAAAAAA-3′

(P3) FAM-labeled C23 DNA, 5′-/56-FAM/CCCCCCCCCCCCCCCCCCCCCCC-3′

(P4) FAM-labeled T23 DNA, 5′-/56-FAM/TTTTTTTTTTTTTTTTTTTTTTT-3′

Carboxyl graphene water dispersion was purchased from ACS Material, Medford, MA 02155, USA and sonicated 12 h before use, which resulted in a highly stable nanosized graphene oxide (nGO). Sodium cholate hydrate was purchased from Alfa Aesar, Tewksbury, MA 01876, USA. Raw molybdenum(iv) sulfide and tungsten(iv) sulfide were purchased from Sigma-Aldrich, St. Louis, MO 63103, USA. Bovine serum albumin (BSA) was purchased from Amresco, Solon, OH 44139, USA. Lipase (from *Candida rugosa*), alkaline phosphatase (from bovine intestinal mucosa), protease (from *Streptomyces griseus*), β-galactosidase (from *Aspergillus oryzae*) and cytochrome c (from equine heart) were purchased from Sigma-Aldrich, St. Louis, MO 63103, USA. Among the breast cancer cell lines, MCF-7 was obtained from Prof. Maksim Royzen at the University at Albany, SUNY; MDA-MB-231 was gifted by Prof. Zdravka Medarova at Harvard Medical School; and BT-20 cell line was purchased from American Type Culture Collection (ATCC), Manassas, VA 20110, USA. Poly(*N*-isopropylacrylamide) (PNIPAM) with 70 kDa molecular weight and lower critical solution temperature (LCST) of 39 °C was provided by Prof. Mustafa S. Yavuz at Selcuk University, TR.^68^ All other reagents were obtained from Sigma-Aldrich, St. Louis, MO 63103, USA and used without further purification. Double distilled water was used in the preparation of all solutions.

### Synthesis of water-soluble nanosized transition metal dichalcogenides (TMDs)

Nanosized TMDs were prepared according to the procedure published in the literature.^69^ Briefly, raw solid MoS_2_ or WS_2_ was mixed with sodium cholate in 5 : 1 (w/w) ratio in 300 mL of water and sonicated for 20 h using the ultrasonic processor (120 W and 20 kHz with pulse-on for 2 s and pulse-off for 4 s) in the ice bath to prevent overheating. The resultant black dispersion was centrifuged at 3000 rpm for 30 min. The yellow-green supernatant containing dispersed TMDs was separated from the precipitate and subjected to another centrifugation using a higher speed at 12 000 rpm for 30 min. The nanosized TMD pellet at the bottom of the tube was redispersed in water and sonicated to extract the intercalated sodium cholate. This new dispersion was centrifuged at 12 000 rpm for 30 min and washed with water. The centrifugation and washing step was repeated at least three times to remove sodium cholate completely. Resulting precipitate was dispersed in 300 mL of deionized water to prepare a suspension of nanosized TMD particles. Hydrodynamic size of the nanosized TMDs was measured using dynamic light scattering (DLS), DynaPro Titan, Wyatt Technology Corporation, USA.

### Preparation of non-specific 2D nanoprobes (2D-nps) for sensor array

The non-specific sensor array in this study is composed of 12 fluorescently silent 2D nanomaterial-DNA nanoassemblies (2D nanoprobes: 2D-nps). The 2D-nps were prepared by incubating the nanoparticles (nGO, MoS_2_ or WS_2_) with 20 nM FAM-labeled DNA molecules for 30 min as described below. The rate and degree of the adsorption were characterized by monitoring the decrease in the fluorescence intensity of the labeled ssDNAs upon incubation with each nanoparticle. The fluorescence of each DNA was quenched by the nanoparticles upon adsorption due to the characteristic quenching properties of each nanoparticle.

#### Protein identification and breast cancer cell identification studies

For 20 nM of each probe DNA (FAM-scrambled, FAM-A23, FAM-C23, FAM-T23); 1.2 μg mL^–1^ final nGO [in 100 mM phosphate buffer (pH 7.2, 150 mM NaCl, 1 mM MgCl_2_)], 15 μg mL^–1^ final MoS_2_ [in 25 mM HEPES buffer (pH 7.5, 100 mM NaCl, 10 mM MgCl_2_)], or 15 μg mL^–1^ final WS_2_ [in 25 mM HEPES buffer (pH 7.5, 100 mM NaCl, 10 mM MgCl_2_)] was used to completely quench the fluorescence during 30 min incubation.

#### Polymer conformation identification study

For 20 nM of each probe DNA (FAM-scrambled, FAM-A23, FAM-C23, FAM-T23); 1.2 μg mL^–1^ final nGO [in 100 mM phosphate buffer (pH 7.2, 150 mM NaCl, 1 mM MgCl_2_)], 15 μg mL^–1^ final MoS_2_ [in 25 mM HEPES buffer (pH 7.5, 100 mM NaCl, 10 mM MgCl_2_)], or 20 μg mL^–1^ final WS_2_ [in 25 mM HEPES buffer (pH 7.5, 100 mM NaCl, 10 mM MgCl_2_)] was used to completely quench the fluorescence during 30 min incubation.

### Adsorption/quenching of ssDNAs using 2D nanoparticles for a model 2D-np

#### Adsorption/quenching study

For 20 nM of P2 (FAM-A23); 3.0 μg mL^–1^ final nGO [in 100 mM phosphate buffer (pH 7.2, 150 mM NaCl, 1 mM MgCl_2_)], MoS_2_ [in 25 mM HEPES buffer (pH 7.5, 100 mM NaCl, 10 mM MgCl_2_)], or WS_2_ [in 25 mM HEPES buffer (pH 7.5, 100 mM NaCl, 10 mM MgCl_2_)] was used to adsorb P2 DNA molecules as a model system *via* 30 min incubation. Rate of fluorescence quenching was recorded by measuring the change in the fluorescence intensities over 1 h.

#### Displacement event

20 nM of P4 (FAM-T23) was used to construct the fluorescently silent 2D-nps. The non-specific desorption studies were carried out using 1 μM final alkaline phosphatase with [nGO-P4], [MoS_2_-P4], [WS_2_-P4] 2D-nps as a model system in 100 μL of reaction buffer. Signal recovery was obtained through 2 h kinetics studies. Experiments were performed in triplicates.

### Protein identification studies

#### Identification of five distinct proteins with constant concentration

Non-specific sensor array composed of 2D-nps was employed to observe the characteristic fluorescence response of five distinct protein molecules with a constant concentration. The aforementioned sensor array composed of 12 distinct 2D-nps was prepared in 100 μL buffers and tested for 1 μM of final BSA, lipase, alkaline phosphatase, protease, or β-galactosidase concentration, respectively. At the end of 2 hour kinetic study, the final fluorescence values were used to calculate the fluorescence recovery (Δ*f* = *f* – *f*_0_) for each protein to obtain a data matrix. Experiments were carried out in six replicates and the fluorescence response patterns were obtained using the data matrix. Later, the experiments were carried out for different concentrations (0.5 μM, 1.0 μM, 2.0 μM, 3.0 μM or 4.0 μM) of an individual protein for constructing concentration-dependent fluorescence response patterns.

#### Identification of five proteins with identical absorption values

The sensor array was tested towards five proteins for their concentrations corresponding to a constant absorbance value of 0.1 a.u. This is a required calibration study that enables one to identify an unknown protein with an unknown concentration as described in the aforementioned results. First, 1 μM of each one of the five proteins were analyzed using UV-visible spectrometer to determine their initial absorbance at 280 nm. Abs_280_ values for 1 μM samples were following; BSA: 0.035, lipase: 0.034, alkaline phosphatase: 0.121, protease: 0.030, β-galactosidase: 0.031 a.u. Afterwards, the protein samples were prepared as either diluted or more concentrated to obtain ∼0.1 a.u.-equivalent concentrations. Then, absorbance measurements were performed for the normalized samples. Normalized Abs_280_ values and the corresponding protein concentrations were determined to be following; 2.9 μM BSA: 0.1, 3.8 μM lipase: 0.09, 0.8 μM alkaline phosphatase: 0.09, 4.4 μM protease: 0.1, 3.2 μM β-galactosidase: 0.09 a.u. These 0.1 a.u.-equivalent protein amounts were tested by the sensor array with 100 μL of 12 2D-nps. At the end of 2 h kinetics, final fluorescence numbers were used to calculate Δ*f* and to create the training matrix for constructing fluorescence response patterns.

#### Unknown protein type identification and concentration detection

The sensor array composed of 12 2D-nps was tested against 15 unknown protein samples derived from 5 standard proteins with unknown concentrations in a double-blinded fashion. Protein types and their original concentrations were not known to the tester until the prediction was done based on the obtained results. Each unknown sample was analyzed using UV-visible spectrometer to determine the initial absorbance. Then, necessary dilution was done to obtain OD value equivalent to 0.1 a.u. and the dilution coefficient was recorded for each sample. These 0.1 a.u.-equivalent concentrations were used as final concentrations in 100 μL of each 2D-np in the sensor array. The fluorescence recovery which was achieved following the displacement of DNA probes was noted at the end of 2 h kinetics. Δ*f* was calculated and the data matrix was produced for prediction. Partial Least Squares (PLS) prediction function was employed for this data to predict the protein type as clusters in which the samples fit according to the results in the calibration data. After identities of the unknown proteins were determined, we used the related dilution coefficients to approximately calculate the original concentrations backwards.

### Breast cancer cell identification studies

MDA-MB-231 and MCF-7 cell lines were cultured in DMEM (Invitrogen) supplemented with 10% fetal bovine serum. BT-20 cell line was cultured in EMEM supplemented with 10% fetal bovine serum. The cells were propagated in media supplemented with 100 U mL^–1^ penicillin and 100 μg mL^–1^ streptomycin (Life Tech Corp., Grand Island, NY) at 37 °C in a 5% CO_2_ incubator.

#### Identification of three cell lines with constant cell count

Three breast cancer cell lines; MDA-MB-231, MCF-7 and BT-20; with different cancer stages and/or cell surface expressions were identified based on the fluorescence response patterns produced by our sensor array against a constant number of cells. Three cell lines were trypsinized and dispersed in 8 mL of phosphate buffer (pH 7.4). After cell counting with hemocytometer, the portion of the cell suspensions including ∼1000 cells was added into 100 μL of each 2D-np suspension in the sensor array. At the end of 2 h kinetics, Δ*f* was determined and the data matrix was created for the fluorescence response patterns. These results were used as the calibration plot for unknown cell identification.

#### Unknown cell type identification

Our sensor array was challenged against 9 unknown cell samples prepared out of three standard cell lines through a blinded test. Cell types were unknown to the tester until the predictions were complete. Three cell lines were trypsinized and dispersed in 9 mL of phosphate buffer (pH 7.4). These 9 mL suspensions were split into 2 mL, 3 mL and 4 mL fractions, each containing different cell amounts. After cell counting was done, corresponding volumes from each unknown containing ∼1000 cells were tested with the sensor array.

At the end of each 2 hour kinetics, final fluorescence was obtained in each 2D-np as a result of DNA displacement induced by the cells. Δ*f* was calculated and the training matrix was prepared for cell identification. PLS prediction function was employed for this data to verify the cell types.

### Conformation study

Two distinct conformations of poly(*N*-isopropylacrylamide) (PNIPAM) polypeptide below and above its lower critical solution temperature (LCST: 39 °C) were discriminated using the non-specific sensor array.

#### Durability test

In order to demonstrate that the fluorescently silent 2D-nps are stable at the temperatures chosen for PNIPAM conformation studies, each 2D-np in the sensor array was incubated with no target at 24° and 45 °C, separately. The change in the fluorescence intensity was monitored for 2 h. At the end, the final fluorescence (*f*) was recorded and compared to the signal at 0 min (*f*_0_) for each set. As expected no significant change was observed. Experiments were carried out in three replicates.

#### Identification of two conformations of the polymer

Two distinct configurations of PNIPAM were identified using the sensor array. PNIPAM undergoes structure-switching, between hydrophilic and hydrophobic states, at its LCST (39 °C) value. 2.5 μL of 1.5 mg mL^–1^ PNIPAM stock (0.037 mg mL^–1^ final) was added into each 100 μL 2D-np well in the sensor array. The fluorescence recovery in each 2D-np was recorded at 24° and 45 °C, separately. Δ*f* was used to prepare the training matrix and fluorescence response pattern of each conformation.

### Fluorescence measurements

Fluorescence measurements were carried out using a BioTek Synergy™ H1 microplate reader. For kinetics measurements for FAM-labeled DNAs, samples were excited at 485 nm and the emission data was collected at 518 nm through non-stop reading with 2 min intervals.

### Statistical analysis

The training data matrices were processed using Partial Least Squares Discriminant Analysis (PLSDA) in PLS Toolbox, in SOLO (version 8.1) and experiments were performed in six replicates to construct the training matrix and fluorescence response patterns. Rest of the data was expressed as mean ± SD.

## Author contributions

MY conceived the study. MY and MSH designed the experiments. MSH performed all of the sensing studies and PLS analysis. MB prepared the two-dimensional nanoparticles. NR performed *in vitro* cell studies. EA and MR helped with the characterization of the nanoparticles. MY and MSH wrote the manuscript.
