# Supplementary material for: Universal sensor array for highly selective system identification using two-dimensional nanoparticles
Source: Chem Sci. 2017 Jun 16;8(8):5735–45. doi: 10.1039/c7sc01522d (PMC5621473; doi:10.1039/c7sc01522d)
Supplement: Supplementary file 1 [file SC-008-C7SC01522D-s001.pdf]

Electronic Supplementary Material (ESI) for Chemical Science.
This journal is © The Royal Society of Chemistry 2017

Supporting Information

Universal sensor array for highly selective system identification using two-dimensional nanoparticles

Mustafa Salih Hizir†, Neil M. Robertson†, Mustafa Balcioglu†, Esma Alp†, Muhit Rana†, and Mehmet V. Yigit*,†,‡

† Department of Chemistry,
University at Albany, State University of New York,
1400 Washington Avenue, Albany, New York 12222, United States.

‡ The RNA Institute,
University at Albany, State University of New York,
1400 Washington Avenue, Albany, New York 12222, United States.

*Correspondence:

Tel: (1) 518-442-3002

myigit@albany.edu

Abstract

Here, we have listed the results of additional experiments, characterization of 2D nanomaterials and their nanoassemblies, and tables for Partial Least Squares (PLS) discriminant analysis as supplementary information for the manuscript.

Table of Content

1. Supporting Figures ………………………S3-S13

1. Supporting Figures.

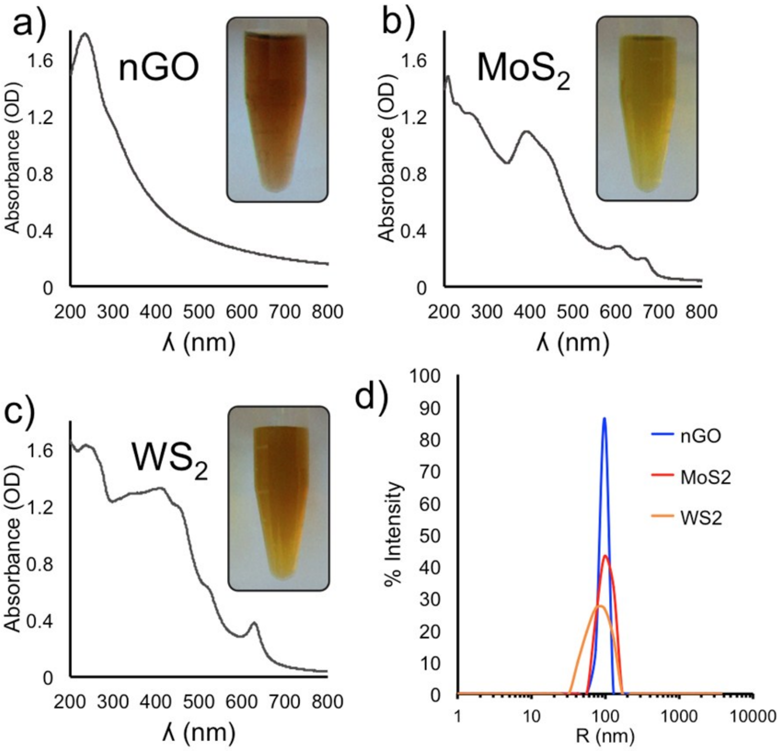

Figure S1: Characterization of 2D nanomaterials and determination of hydrodynamic size. Absorbance spectra demonstrating (a) nGO (b) MoS_2 (c) WS_2 characteristic peaks. Insets are water-soluble nGO, MoS_2, and WS_2 images, respectively. (d) Hydrodynamic radius of nGO, MoS_2, or WS_2 was determined using dynamic light scattering.

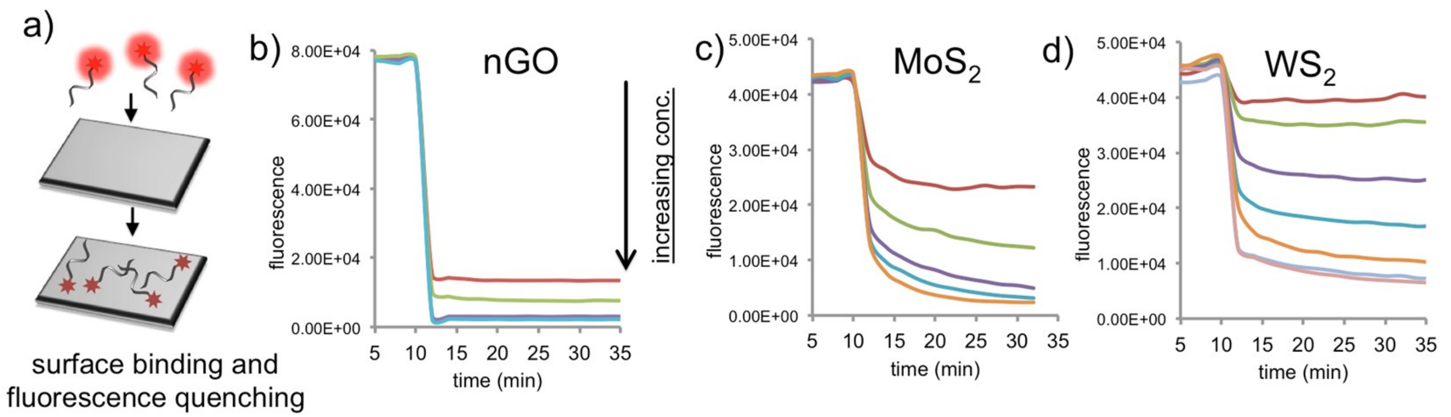

Figure S2: a) Schematic of adsorption and fluorescence-quenching of a fluorescent DNA probe on 2D nanoparticles (nGO, MoS_2, WS_2). Adsorption of a fluorescent DNA probe with increasing concentrations of a) nGO, b) MoS_2 and c) WS_2.

Protein	n	np-1	np-2	np-3	np-4	np-5	np-6	np-7	np-8	np-9	np-10	np-11	np-12
BSA	1	3655	6326	676	4999	1737	3193	421	1875	370	297	207	326
BSA	2	3604	6332	646	5059	1869	2753	370	1455	350	1172	189	420
BSA	3	3413	6194	770	5165	1681	2792	429	1485	324	289	192	467
BSA	4	3454	6193	671	4927	1395	2660	395	1582	321	379	135	472
BSA	5	3348	5977	649	4957	1669	2354	423	1844	405	275	144	371
BSA	6	3313	5825	697	4923	1531	2752	393	1428	495	530	188	157
Lipase	1	5303	8956	1815	6117	2190	4309	862	1674	427	535	224	300
Lipase	2	5315	9213	1694	6212	2141	3831	638	1549	330	535	284	389
Lipase	3	5230	9183	1537	6115	2014	3903	612	1539	467	427	233	309
Lipase	4	5175	8850	1489	6000	1846	3677	686	1415	341	339	238	185
Lipase	5	5214	8833	1458	6171	1869	3925	601	1463	364	323	263	200
Lipase	6	4879	8520	1527	5929	1807	3794	686	1479	333	391	188	173
Phosphatase Alkaline	1	6629	10206	2140	10138	7284	7926	2403	6549	2109	2353	964	2434
Phosphatase Alkaline	2	6462	10080	2106	10149	7390	8521	2674	6529	2275	3064	1032	2906
Phosphatase Alkaline	3	6275	10152	2120	10108	7281	8357	2842	6486	2250	3173	1022	3130
Phosphatase Alkaline	4	6376	9985	2165	10258	7748	8141	2754	6486	2103	2788	994	2919
Phosphatase Alkaline	5	6394	9685	2062	10035	7278	8548	2779	7289	2218	2745	1124	2993
Phosphatase Alkaline	6	5999	9282	2060	9939	7262	8766	2968	6435	2425	3064	1010	3190
Protease	1	96	143	-3	212	-33	-519	-12	-58	11	-27	-40	-129
Protease	2	15	96	72	329	-42	-808	-67	-41	-21	-152	-72	-35
Protease	3	137	110	25	262	-86	-854	-7	-104	-52	424	-44	-137
Protease	4	43	161	58	300	-97	-836	-76	-99	-48	-129	-112	-214
Protease	5	158	88	90	220	-51	-648	-88	-6	37	-223	-62	-196
Protease	6	19	111	97	225	-54	-708	-107	-27	-59	-282	-53	-167
Beta-galactosidase	1	1469	3450	243	2747	-66	-446	-39	-172	-43	-207	-90	-223
Beta-galactosidase	2	1074	3136	197	2833	-52	-365	-69	42	32	-273	-103	-210
Beta-galactosidase	3	1102	3169	251	2844	-43	-472	-78	-27	-29	-252	-65	-191
Beta-galactosidase	4	1196	3252	245	2856	-5	-467	-148	-34	-60	-386	-66	-290
Beta-galactosidase	5	1175	3076	177	2927	-78	-358	-127	-22	-59	-393	-40	-254
Beta-galactosidase	6	1131	3158	327	2922	-95	-389	-17	-20	-41	-280	8	-244

Figure S3: Training matrix of the fluorescence response patterns of the nanoprobe sensor array (np1-np12) against various proteins with a constant 1 µM concentration.

Protein	n	np-1	np-2	np-3	np-4	np-5	np-6	np-7	np-8	np-9	np-10	np-11	np-12
BSA	1	4596	8057	1418	5700	1796	2924	599	1709	625	960	251	699
BSA	2	4415	7734	1382	5660	2081	2928	698	1706	688	1696	295	706
BSA	3	4587	7864	1428	5684	1945	3057	691	1564	576	1305	246	636
BSA	4	4499	7929	1343	5844	1722	2741	556	1757	381	609	227	618
BSA	5	4386	7662	1280	5727	1787	2654	643	2088	502	730	223	908
BSA	6	4339	7713	1380	5700	1893	2423	702	1909	405	609	261	420
Lipase	1	7971	11833	2698	7498	2888	4993	1075	2372	447	523	291	259
Lipase	2	8063	11951	2611	7555	3132	5063	1049	2476	527	495	274	220
Lipase	3	7980	11664	2626	7308	2890	4976	1061	2323	460	543	228	406
Lipase	4	8322	12391	2812	7551	3287	5354	1209	2822	413	618	309	335
Lipase	5	8088	11963	2889	7525	3300	5267	1309	2718	432	529	203	369
Lipase	6	8080	11861	2886	7503	3176	5160	1208	2728	450	394	252	220
Phosphatase Alkaline	1	5605	9354	1812	8727	6634	8653	2320	6339	1900	2483	909	3106
Phosphatase Alkaline	2	5360	9123	1838	8988	6650	8181	2147	6882	1944	2453	787	2541
Phosphatase Alkaline	3	5440	9174	1807	8802	6376	7673	2209	6724	1984	2625	845	2598
Phosphatase Alkaline	4	5622	9061	1805	8975	6228	7640	2058	6543	1944	2517	940	2438
Phosphatase Alkaline	5	5513	9162	1839	8695	6857	7688	2300	7117	2070	2874	797	2604
Phosphatase Alkaline	6	5045	8895	1856	8905	6593	8172	2056	6458	2170	3081	878	2683
Protease	1	485	1963	249	1644	27	276	-38	-24	180	-157	38	-153
Protease	2	420	1969	322	1644	42	174	19	-77	60	-228	10	-126
Protease	3	436	1925	243	1678	96	71	14	44	105	-202	-28	-136
Protease	4	415	1988	231	1728	60	60	-54	8	23	-347	-7	-59
Protease	5	435	1916	324	1672	47	120	39	-14	38	-125	45	-98
Protease	6	403	2030	120	2025	24	134	-22	22	31	229	29	-158
Beta-galactosidase	1	6178	9286	1765	7491	1263	2560	277	1361	520	955	273	662
Beta-galactosidase	2	5926	9184	1625	7293	1267	2618	227	1368	499	1403	254	530
Beta-galactosidase	3	5974	9213	1759	7303	1055	2656	169	1396	585	945	269	763
Beta-galactosidase	4	5936	8836	1708	7170	1392	2780	275	1388	515	1064	246	1119
Beta-galactosidase	5	6073	8949	1738	7224	1235	2691	237	1600	446	866	244	846
Beta-galactosidase	6	5848	8712	1871	7325	1181	2615	274	1451	470	941	201	632

Figure S4: Training matrix of the fluorescence response patterns of the nanoprobe sensor array (np1-np12) against various proteins with identical absorbance values (Abs_{280} = 0.1).

Protein	n	np-1	np-2	np-3	np-4	np-5	np-6	np-7	np-8	np-9	np-10	np-11	np-12
0.5 µM	1	1201	2884	277	3195	596	1365	169	559	109	79	121	430
0.5 µM	2	1182	2770	362	3045	532	1238	163	559	110	176	170	226
0.5 µM	3	1132	3094	295	3166	494	1341	110	462	123	215	221	206
0.5 µM	4	1136	2902	327	3116	442	1334	150	578	121	199	107	184
0.5 µM	5	1077	3019	262	3180	439	1290	196	517	123	168	93	205
0.5 µM	6	1252	3027	225	3317	429	1343	191	676	68	167	136	252
1 µM	1	1598	3941	429	3602	710	1752	387	728	110	149	148	308
1 µM	2	1625	3732	453	3608	750	1798	375	726	118	264	162	307
1 µM	3	1658	3890	417	3554	684	1807	236	648	159	297	185	261
1 µM	4	1588	3693	416	3704	660	1684	267	731	128	265	142	211
1 µM	5	1545	3832	447	3903	618	1690	281	742	157	255	151	263
1 µM	6	1577	3847	428	4145	650	1702	291	784	138	279	116	249
2 µM	1	2457	4908	650	4227	1006	2293	368	1070	174	294	196	347
2 µM	2	2492	4982	669	4200	952	2288	407	1016	188	293	198	319
2 µM	3	2483	4898	655	4377	977	2271	295	1042	211	403	259	345
2 µM	4	2366	4979	588	4292	887	2338	396	1118	162	410	188	390
2 µM	5	2328	5286	649	4510	954	2091	368	1081	179	398	183	371
2 µM	6	2389	4793	610	4969	1000	2263	366	1039	154	359	235	417
3 µM	1	3811	5798	840	4782	1392	2610	571	1267	166	481	303	553
3 µM	2	3842	5501	843	4747	1226	2668	526	1164	234	447	253	444
3 µM	3	3877	5997	776	4804	1261	2699	493	1147	275	524	283	499
3 µM	4	3881	5899	790	4834	1145	2608	591	1226	229	497	239	494
3 µM	5	3784	5836	842	4945	1137	2707	482	1268	220	753	233	637
3 µM	6	3794	6121	896	5714	1159	2712	531	1690	229	811	268	827
4 µM	1	4750	8375	1207	5135	1634	2930	595	1569	237	916	315	814
4 µM	2	4756	8280	1171	4874	1555	2982	613	1499	274	918	301	1120
4 µM	3	4897	8884	1194	4954	1525	3048	580	1472	392	1200	311	1294
4 µM	4	5061	8764	1147	5407	1480	2998	621	1472	340	1228	384	1002
4 µM	5	5100	9250	1198	5787	1381	3036	537	1495	450	1222	327	1274
4 µM	6	5240	9658	1162	6257	1407	2950	581	1684	473	1269	425	1474

Figure S5: Training matrix of the fluorescence response patterns of the nanoprobe sensor array (np1-np12) against various concentrations of a protein.

Cell lines	n	np-1	np-2	np-3	np-4	np-5	np-6	np-7	np-8	np-9	np-10	np-11	np-12
MDA-MB-231	1	48	261	115	341	65	330	-7	75	425	1022	296	689
MDA-MB-231	2	48	552	99	286	38	176	-46	101	377	1041	303	665
MDA-MB-231	3	94	179	162	214	64	284	5	118	408	1089	360	707
MDA-MB-231	4	46	261	134	178	50	82	80	47	323	1238	366	898
MDA-MB-231	5	101	285	120	273	63	39	34	47	435	1176	367	792
MDA-MB-231	6	44	244	118	343	48	180	-10	100	1223	1904	452	1106
MCF-7	1	6055	10519	2435	7448	3701	5988	818	3277	1847	4191	861	2062
MCF-7	2	6324	11292	2158	7618	3930	5555	686	3210	1619	4759	882	2537
MCF-7	3	6459	10940	2398	7857	4546	6025	1160	3799	1431	4081	890	1804
MCF-7	4	6700	10982	2443	7928	4388	6717	1059	3660	1854	4644	1082	1916
MCF-7	5	6602	11063	2590	7968	4591	6290	1015	4032	1648	4680	836	2769
MCF-7	6	6844	10968	2513	8030	4620	6843	1166	3856	1645	3590	927	2415
BT-20	1	2483	6488	689	4572	1661	2620	223	1366	506	1408	511	1041
BT-20	2	2558	6688	627	4480	1268	2516	230	1342	594	1422	510	1387
BT-20	3	2335	6106	640	4886	1691	2498	229	1178	561	1453	654	1628
BT-20	4	2585	6509	596	4893	1666	3085	299	1129	542	1485	652	1900
BT-20	5	2691	6312	548	4869	1322	2430	284	1210	808	2100	637	1891
BT-20	6	2761	6347	559	5397	1355	3011	234	1270	499	2712	757	1758

Figure S6: Training matrix of the fluorescence response patterns of the nanoprobe sensor array (np1-np12) against various breast cancer cell types with a constant cell number (~1000 cells).

conformation	n	np-1	np-2	np-3	np-4	np-5	np-6	np-7	np-8	np-9	np-10	np-11	np-12
hydrophilic	1	-35	-28	29	-28	3161	4312	776	3566	1425	2942	414	2829
hydrophilic	2	40	25	5	-34	3124	4142	795	3760	952	2206	433	2662
hydrophilic	3	-4	3	-3	63	3271	4523	751	3603	922	2607	516	2460
hydrophilic	4	11	7	-1	40	2784	4092	792	3437	798	2353	385	2226
hydrophilic	5	-3	-10	78	-15	2828	3865	834	3474	1108	2032	346	2263
hydrophilic	6	-20	3	-49	55	2876	4223	838	3434	1026	2422	433	1939
hydrophobic	1	87	93	3	65	6575	6811	5090	7728	3190	3650	2321	4663
hydrophobic	2	32	158	7	10	6388	7091	5268	7598	2973	3830	2134	4821
hydrophobic	3	82	117	-27	73	7056	6837	4933	7558	2311	3056	2095	4508
hydrophobic	4	32	76	-8	52	6416	6753	5052	7638	2308	2641	2105	4350
hydrophobic	5	72	164	66	6	6559	6761	4969	7446	2311	3016	2142	4042
hydrophobic	6	65	134	114	85	6472	6826	4948	6898	2261	3285	2233	4547

Figure S7: Training matrix of the fluorescence response patterns of the nanoprobe sensor array (np1-np12) against distinct molecular PNIPAM configurations.

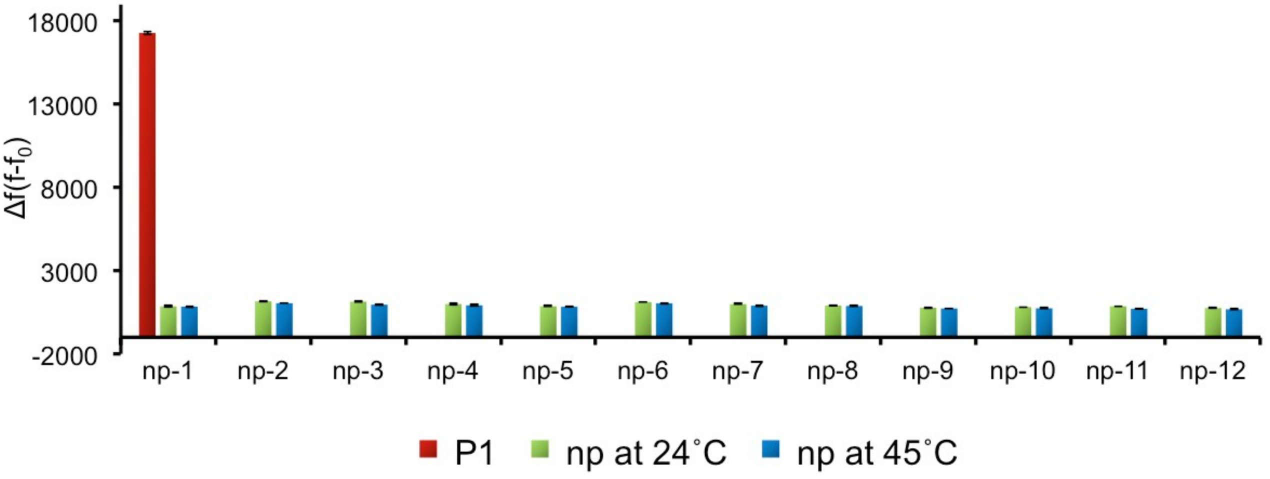

Figure S8: Durability test demonstrating that all nanoprobe assemblies are identically stable at 25 and 45 ˚C and generate almost no false signal, Δf ($f - f_0$), over a 2-hour time period. The Δf (blue and green bars) in each system is also compared to the fluorescence of the DNA probe (red bar).

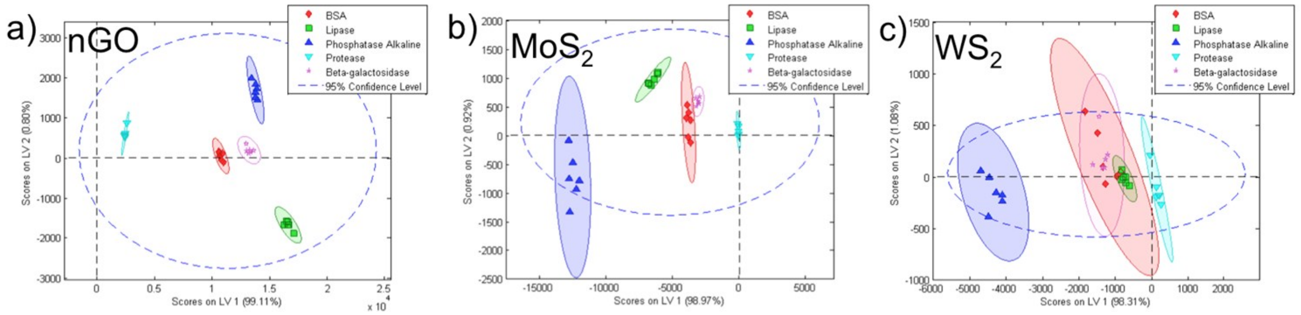

Figure S9: Canonical score plots for the first two latent variables of processed fluorescence response patterns of monotypic (a) nGO (np1-np4), (b) MoS_2 (np5-np8), (c) WS_2 (np9-np12) sensor arrays obtained against the proteins (BSA, Lipase, Alkaline phosphatase, Protease, β-galactosidase) with identical absorbances (0.1 a.u.). The canonical scores were calculated by PLS for absorbance-normalized proteins. Individual sensor designs ended up with poor discrimination and profound interference.

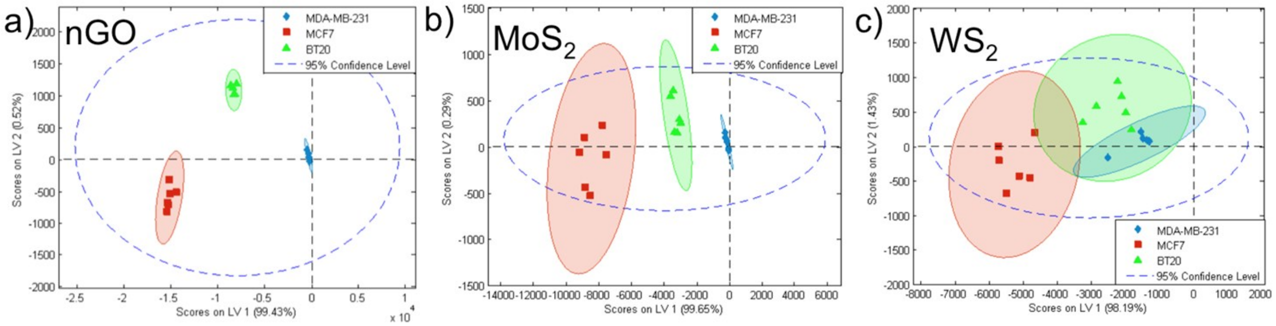

Figure S10: Canonical score plots for the first two latent variables of processed fluorescence response patterns of monotypic (a) nGO (np1-np4), (b) MoS_2 (np5-np8), (c) WS_2 (np9-np12) sensor arrays obtained against the breast cancer cell lines (MDA-MB-231, MCF-7, BT-20) with a constant count (~1000 cells). The canonical scores were calculated by PLS for three cell types. Individual sensor designs ended up with poor discrimination and profound interference.

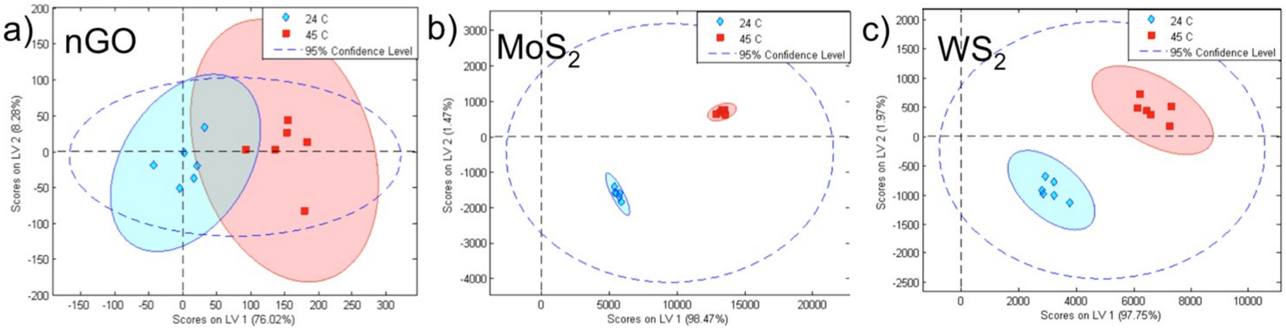

Figure S11: Canonical score plots for the first two latent variables of processed fluorescence response patterns of monotypic (a) nGO (np1-np4), (b) MoS_2 (np5-np8), (c) WS_2 (np9-np12) sensor arrays obtained against the two conformations of PNIPAM (hydrophilic/soluble and hydrophobic/insoluble). The canonical scores were calculated by PLS for two polymer states. Individual sensor designs ended up with poor discrimination and profound interference.
